# Impact of rs2414096 polymorphism of *CYP19* gene on susceptibility of polycystic ovary syndrome and hyperandrogenism in Kashmiri women

**DOI:** 10.1038/s41598-021-92265-1

**Published:** 2021-06-21

**Authors:** Sairish Ashraf, Shayaq Ul Abeer Rasool, Mudasar Nabi, Mohd Ashraf Ganie, Shariq R. Masoodi, Shajrul Amin

**Affiliations:** 1 Department of Biochemistry, University of Kashmir, Srinagar, 190006 India; 2 Department of Biotechnology, University of Kashmir, Srinagar, India; 3 Department of Endocrinology, Sher-i-Kashmir Institute of Medical Sciences (SKIMS), Srinagar, India

**Keywords:** Biochemistry, Genetics, Endocrinology

## Abstract

Polycystic ovary syndrome (PCOS) is the most common reproductive endocrine disorder in pre-menopausal women having complex pathophysiology. Several candidate genes have been shown to have association with PCOS. *CYP19* gene encodes a key steroidogenic enzyme involved in conversion of androgens into estrogens. Previous studies have reported contradictory results with regard to association of SNP rs2414096 in *CYP19* gene with PCOS and hyperandrogenism in different ethnic populations. Present study was aimed to investigate the impact of SNP rs2414096 polymorphism of *CYP19* gene on susceptibility of PCOS and hyperandrogenism in Kashmiri women. Further we also studied the genotypic-phenotypic association for various clinical and biochemical parameters of this polymorphism. Case control study. 394 PCOS cases diagnosed on the basis of Rotterdam criteria and age matched 306 healthy women. We found a significant differences in genotypic frequency (χ^2^ = 18.91, p < 0.05) as well as allele frequency (OR 0.63, CI 0.51–0.78, χ^2^ = 17.66, p < 0.05) between PCOS women and controls. The genotype–phenotype correlation analysis showed a significant difference in FG score (p = 0.047) and alopecia (p = 0.045) between the three genotypes. Also, the androgen excess markers like DHEAS (p < 0.001), Androstenedione (p < 0.001), Testosterone (p < 0.001) and FAI (p = 0.005) were significantly elevated in GG genotype and showed a significant difference in additive model in PCOS women. rs2414096 polymorphism of *CYP19* gene is associated with the risk of PCOS as well as with clinical and biochemical markers of hyperandrogenism, hence suggesting its role in clinical manifestations of PCOS in Kashmiri women.

## Introduction

Polycystic ovarian syndrome is a heterogeneous, complex and polygenic disorder in reproductive-aged women having diverse implication including endocrine, reproductive, dermatological and metabolic and psychological abnormalities^1^. PCOS women have a higher risk of various complications like insulin resistance, diabetes, cardiovascular problems and carcinomas of ovary, uterus and ovary^2^. The worldwide prevalence of PCOS is around 5–10%^3^. One of the most consistent and salient feature of PCOS is production of excess amounts of androgens leading to hyperandrogenism, which is clinically manifested as hirsutism, acne, alopecia or biochemically as increased testosterone levels, thus implicating defects in the steroid synthesis pathway. Genetic variants of various genes like *CYP17, CYP11, CYP19* involved in steroid pathway are proposed to be involved in the development of the hyperandrogenism in PCOS.

*CYP19* gene is positioned on the long arm of chromosome 15 (15q21.1)^4^. It contains ten exons spanning over a region of 123 kb and encodes a key steroidogenic enzyme known as aromatase (P450arom) which is a member of the cytochromeP450 family of enzymes^3^. This enzyme complex includes cytochrome P450 aromatase (P450arom) as well as Nicotinamide Adenine Dinucleotide Phosphate (NADPH) and cytochrome P450 reductase. Aromatase is expressed in estrogen producing tissues which includes ovaries, placenta, testes, adipose tissue, bone, brain, skin and vascular smooth muscle cells^5^. In reproductive aged-women, production of aromatase from ovarian granulosa plays an important role. This enzyme catalyzes the final step of estrogen biosynthesis wherein testosterone and androstenedione are converted to estradiol and estrone respectively^6^, hence may play a significant role in the development of hyperandrogenism. It has been documented that PCOS women contain low levels of aromatase in their granulosa cells obtained from medium-sized follicles^7^. Similarly, it was demonstrated by Jakimiuk et al. that the follicles of PCOS contain insufficient amount of aromatase, low levels of P450arom mRNA and hence low levels of estrogen as compared to control follicles^8^. In an experimental model of ArKO mice, Britt et al. demonstrated that targeted disruption of *CYP19* gene caused formation of large hemorrhagic cystic follicles^9^. Further, Risma et al. proposed the development of ovarian cysts due to abnormally high levels of luteinizing hormone (LH) in ArKO mice models that were aromatase deficient^10^. This deficiency or reduced activity of aromatase in the ovarian follicles and the resulting androgen excess might contribute to the abnormal development of follicles resulting in polycystic ovaries.

Several studies have reported association of single nucleotide polymorphisms (SNPs) rs2414096 in an intronic region of *CYP19* gene with increased androgen concentrations in women with PCOS, thus indicating altered regulation of aromatase involved in PCOS^11, 12^. Previous studies have found single nucleotide polymorphism rs2414096 of *CYP19* to be associated with PCOS and its related phenotypes^13–18^ but other studies did not found any such association of *CYP19* gene in women with PCOS^19, 20^. Only two studies have also been carried out in India wherein one study concluded that variants of *CYP19* gene are significantly associated with PCOS^21^, whereas other study investigating the polymorphism of *CYP19* did not reveal any evidence of the gene being involved in the etiology of PCOS^22^. Recently, a meta-analysis carried out Sharma et al.^23^ on 7 case control studies that included 1414 cases and 1276 controls concluded that rs2414096 polymorphism of CYP19 is associated with the risk of PCOS.

In view of these contradictory results, we designed a case–control study wherein we investigated the impact of rs2414096 single nucleotide polymorphism of *CYP19* on susceptibility of development of PCOS and evaluate genotypic correlation with various features of hyperandrogenism in ethnic Kashmiri population.

## Results

### Basic clinical characteristics of PCOS women vs healthy controls

The baseline anthropometric, biochemical and hormonal parameters of PCOS women and healthy controls are given in Table 1. The mean age, height and mean age of menarche were comparable but features like Body mass index (BMI), Waist-Hip ratio (WHR) and Ferriman-Gallwey (FG score) were higher in cases as compared to controls and was found to be statistically significant (p < 0.001). There was a significant difference between mean fasting glucose and glucose 2 h in cases and healthy controls (p < 0.001). Compared with healthy controls, women with PCOS had statistically significantly increased cholesterol, triglycerides, HDL-C and LDL-C levels (p < 0.001). The levels of urea, creatinine, liver marker enzymes (ALT and AST) were significantly higher in cases than healthy control group. Mean LH levels, FSH levels and LH:FSH values between cases and controls varied significantly (p < 0.001). Mean levels of testosterone, SHBG, DHEA-S and androstenedione also varied significantly among PCOS women and control group (p < 0.001). FAI levels were also found to be statistically higher in cases as compared to controls (p < 0.001). The mean levels of fasting insulin, HOMA-IR, FGIR and QUICKI also varied significantly between PCOS women as compared to healthy women (p < 0.001).

**Table 1:** Anthropometric data of study participants.

Clinical parameter	PCOS (n = 394)	Control (n = 306)	p value
Age (years)	23.06 ± 4.99	22.53 ± 3.22	0.212
Weight (kg)	60.10 ± 11.2	52.19 ± 6.13	< 0.0001*
Height (m)	1.61 ± 0.70	1.57 ± 0.05	0.2848
Waist (cm)	86.63 ± 10.82	78.69 ± 7.36	< 0.0001*
Hip (cm)	94.72 ± 8.72	90.18 ± 6.43	< 0.0001*
W/H ratio	0.91 ± 0.06	0.87 ± 0.06	< 0.0001*
BMI (kg/m^2^)	24.17 ± 4.49	21.17 ± 2.19	< 0.0001*
FG Score	13.55 ± 5.7	5.01 ± 1.65	< 0.0001*
Menarche (y)	13.11 ± 1.08	13.23 ± 1.06	0.1429
LH (IU/L)	8.96 ± 4.39	6.04 ± 2.35	< 0.0001*
FSH (IU/L)	6.13 ± 2.01	7.08 ± 2.00	< 0.0001*
Testosterone (ng/dL)	59.62 ± 18.35	30.08 ± 12.66	< 0.0001*
SHBG (nmol/L)	41.43 ± 18.31	57.73 ± 17.95	< 0.0001*
Androstenedione (ng/mL)	3.35 ± 1.00	2.05 ± 0.89	< 0.0001*
DHEAS (ng/mL)	3.79 ± 1.19	2.22 ± 0.847	< 0.0001*
LH:FSH	1.57 ± 0.83	0.89 ± 0.40	< 0.0001*
FAI	6.06 ± 4.53	1.97 ± 1.14	< 0.0001*
Fasting glucose (mg/dL)	87.38 ± 8.87	84.58 ± 7.59	< 0.001*
Glucose 2 h (mg/dL)	115.23 ± 16.02	101.72 ± 8.95	< 0.001*
HDL	42.34 ± 3.03	45.24 ± 3.32	< 0.001*
LDL	103.47 ± 26.37	85.99 ± 22.15	< 0.001*
Cholesterol (mg/dL)	171.96 ± 25.79	152.08 ± 19.52	< 0.002*
Triglycerides (mg/dL)	131.50 ± 25.17	105.71 ± 13.80	< 0.001*
Urea (mg/dL)	22.70 ± 5.86	21.02 ± 3.98	< 0.001*
Creatinine(mg/dL)	0.94 ± 0.51	0.791 ± 0.14	< 0.001*
AST (U/L)	28.49 ± 10.74	19.68 ± 7.54	< 0.001*
ALT (U/L)	30.43 ± 13.15	24.16 ± 6.00	< 0.001*
Fasting insulin (μIU/mL)	17.56 ± 5.48	12.31 ± 4.36	< 0.001*
HOMA-IR	3.82 ± 1.38	2.58 ± 0.99	< 0.001*
QUICKI	0.32 ± 0.02	0.34 ± 0.02	< 0.001*
FGIR	5.83 ± 4.93	8.02 ± 3.93	< 0.001*

Data presented as mean ± SD. *p-value < 0.05 significant. p values calculated by Independent Student’s t test.

*PCOS* polycystic ovary syndrome, *BMI* body mass index, *FG Score* Ferriman Gallwey Score, *LH* luteinizing hormone, *FSH* follicle stimulating hormone, *SHBG* sex hormone binding globin, *DHEAS* dihydroepiandrostenedione sulphate, *HOMA IR* homeostasis model assessment-estimated insulin resistance, *QUICKI* quantitative insulin sensitivity check index, *FAI* free androgen index, *AST* aspartate aminotransferase, *ALT* alanine aminotransferase.

### Distribution of SNP (rs2414096) of *CYP19* genotypes and alleles

**Figure 1:**
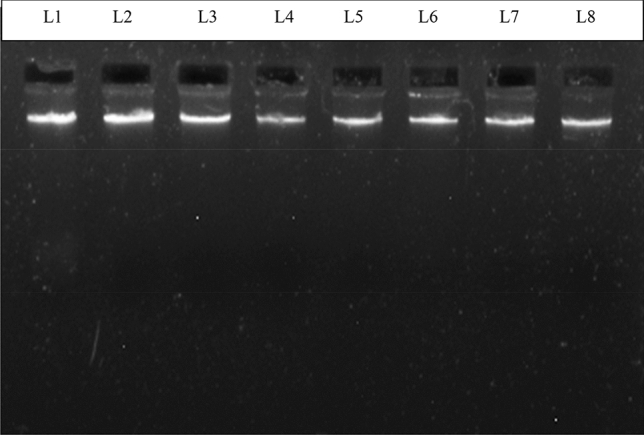
Representative picture of the genomic DNA run on 1% agarose gel.

For genetic analysis DNA was extracted from peripheral blood leukocytes (Fig. 1).

**Figure 2:**
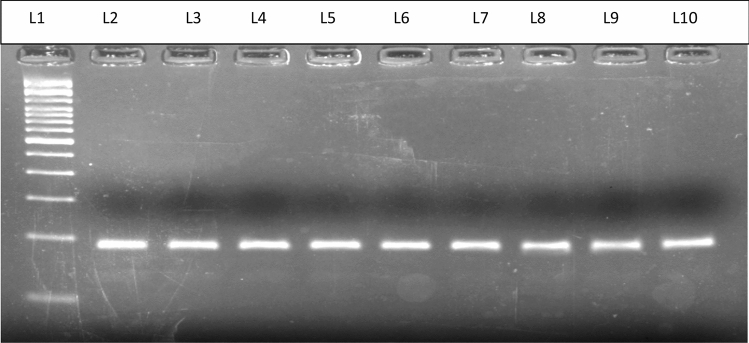
Representative picture of PCR amplification of CYP19 gene. L1 represents 100 bp ladder; L2–L10 show amplified 189 bp PCR product.

All the subjects were then screened for G/A polymorphism in the *CYP19* gene. The region containing G/A SNP (rs2414096) was amplified that resulted in a 189 bp fragment (Fig. 2).

**Figure 3:**
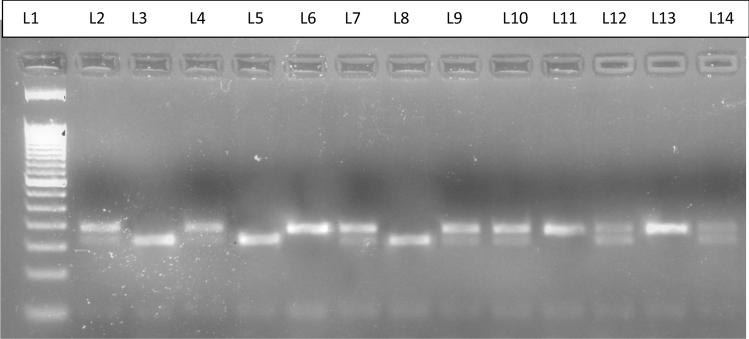
Representative picture for Restriction digestion of CYP19 with CviAII. L1 represents 50 bp ladder; L6, L11, L13-represent wild genotype (189 bp); L3, L5, L8-represents mutant genotype (161 and 28 bp); L2, L4, L7, L9, L10, L12, L14-represents heterozygous genotype (189, 161 and 28 bp).

After the amplification, restriction digestion was performed by CviAII enzyme. The wild type genotype (GG) was not digested yielding a single band of 189 bp; the heterozygote genotype (AG) yielded 3 bands of 189, 161, and 28 bp and homozygous mutant (AA) yielded two fragments of 161 and 28 bp (Fig. 3).

Different models of inheritance were analyzed for the polymorphisms on the basis of Akaike Information Criterion (AIC) which are summarized in Table 2. We found that Log additive model had the least AIC value and hence was found to be best genotypic model for our study.

**Table 2:** Genotypic association between PCOS and control women based on different inheritance model using Akaike Information Criterion (AIC).

Model	Genotype	Cases (n = 394)	Controls (n = 306)	OR (95% CI)	p-value	AIC
Codominant	A/A	79 (20.1%)	91 (29.7%)	1.00	1e−04	946
	G/A	197 (50%)	163 (53.3%)	0.72 (0.50–1.04)		
	G/G	118 (29.9%)	52 (17%)	0.38 (0.25–0.60)		
Dominant	A/A	79 (20.1%)	91 (29.7%)	1.00	0.0031	954.6
	G/A-G/G	315 (80%)	215 (70.3%)	0.59 (0.42–0.84)		
Recessive	A/A-G/A	276 (70%)	254 (83%)	1.00	1e−04	947.2
	G/G	118 (29.9%)	52 (17%)	0.48 (0.33–0.69)		
Overdominant	A/A-G/G	197 (50%)	143 (46.7%)	1.00	0.39	962.6
	G/A	197 (50%)	163 (53.3%)	1.14 (0.85–1.54)		
Log-additive	–	–	–	0.62 (0.50–0.78)	< 0.0001	945

Further, the genotypic and allelic frequencies are summarized in Table 3. Genotypes frequency distribution for rs2414096 polymorphism were in Hardy–Weinberg equilibrium in PCOS (p = 0.844), controls (p = 0.147) and overall (p = 0.45). Among PCOS patients, wild homozygous (GG) were found in 29.9% whereas 50.0% were heterozygous (AG). Among the controls, wild homozygous genotype (GG) was found in 17.0% and heterozygous genotypes (AG) was found in 53.3% of the subjects. The homozygous variant (AA) present in our population was 20.1% in PCOS patients and 29.7% in control population. We found G allele was present in 54.9% PCOS cases and in 43.6% controls whereas A allele was found to be present in 45.1% PCOS cases and 56.4% controls. The allele frequency was found to be significant on comparison between cases and controls (χ^2^ = 17.66; p = 0.0005).

**Table 3:** Genotypic and allelic frequency of *CYP19* gene in PCOS as compared with control women.

		Cases (n = 394)	Controls (n = 306)	OR (95% CI)	X^2^	p-value
Genotypic frequency	GG	118 (29.9%)	52 (17.00%)	–	18.91	0.0005*
	AG	197 (50.0%)	163 (53.3%)			
	AA	79 (20.1%)	91 (29.7%)			
Allelic frequency	G	433 (54.9%)	267 (43.6%)	0.63 0.51–0.78	17.66	0.0005*
	A	355 (45.1%)	345 (56.4%)			

Data is presented as number (%) of PCOS and controls. GG, AG and AA are genotypes in PCOS and controls, *PCOS* polycystic ovary syndrome, G and A are alleles for polymorphism, *OR* Odds Ratio, *CI* confidence interval, χ^2^ chi-square test.

*Significant p value < 0.05.

### Effect of SNP rs2414096 polymorphism of *CYP19* on clinical, hormonal and biochemical parameters

The effect of genetic variants of *CYP19* gene on different clinical, hormonal and biochemical parameters in PCOS cases and control women was analyzed using log-additive model. Table 4 shows the genotype–phenotype correlation within the different genotypes in PCOS women (GG vs GA vs AA). Although we found no overall significant difference between any tested clinical, biochemical and hormonal parameters, a significant association between DHEAS, androstenedione, testosterone and FAI between in GG vs GA, GG vs AA and GA vs AA genotypes was found.

**Table 4:** Clinical characteristics, hormonal and biochemical profile of Log-additive genotype model in PCOS women.

Symptoms	GG (118)	GA (n = 197)	AA (n = 79)	p value
FG Score	13.80 ± 5.715	13.77 ± 5.425	12.62 ± 5.695	0.250
Age	23.728 ± 4.20	23.23 ± 4.458	22.43 ± 4.471	0.127
WHR	0.918 ± 0.061	0.906 ± 0.087	0.904 ± 0.057	0.265
BMI	24.77 ± 4.58	23.86 ± 4.77	23.76 ± 4.00	0.175
Insulin	17.51 ± 5.45	17.37 ± 5.62	18.10 ± 5.18	0.600
DHEAS	4.35 ± 1.24^a,b^	3.67 ± 1.07	3.22 ± 1.02	< 0.001*
Androstenedione	4.01 ± 1.03^a,b^	3.37 ± 0.99^c^	2.64 ± 0.83	< 0.001*
SHBG	41.74 ± 17.60	41.26 ± 16.79	41.36 ± 22.69	0.970
Testosterone	71.27 ± 15.33^a,b^	58.99 ± 16.43^c^	43.74 ± 14.24	< 0.001*
LH	8.95 ± 4.94	9.21 ± 4.07	8.33 ± 4.25	0.327
FSH	5.99 ± 2.01	6.29 ± 2.02	5.91 ± 1.95	0.255
Fasting glucose	87.46 ± 9.04	87.75 ± 8.27	86.71 ± 9.72	0.600
Cholesterol	174.17 ± 25.86	171.20 ± 27.18	170.51 ± 21.92	0.527
Triglycerides	130.07 ± 22.68	130.44 ± 25.39	136.26 ± 27.78	0.170
FAI	6.92 ± 2.98^b^	6.05 ± 5.63	4.79 ± 2.74	0.005*
FGIR	5.63 ± 3.22	6.11 ± 6.26	5.38 ± 2.76	0.473
HOMA-IR	3.78 ± 1.37	3.79 ± 1.40	3.94 ± 1.33	0.690
QUICKI	0.318 ± 0.019	0.318 ± 0.022	0.315 ± 0.017	0.419

Data presented as mean ± SD. *p-value < 0.05 significant. p values calculated by one way Analysis of Variance (ANOVA) independent standard weighted-means analysis and intergroup association tested by Post hoc Tukey HSD test.

*PCOS* polycystic ovary syndrome, *BMI* body mass index, *FG Score* Ferriman Gallwey Score, *LH* luteinizing hormone, *FSH* follicle stimulating hormone, *SHBG* sex hormone binding globin, *DHEAS* dihydroepiandrostenedione sulphate, *HOMA IR* homeostasis model assessment-estimated insulin resistance, *QUICKI* quantitative insulin sensitivity check index, *FAI* free androgen index, *FGIR* fasting glucose insulin ratio.

^a^GG vs GA p < 0.05.

^b^Represents GG vs AA p < 0.05.

^c^Represents GA vs AA p < 0.05.

### Effect of *CYP19* gene polymorphism on hyperandrogenism

The effect of this polymorphism on the various parameters of hyperandrogenism was also analyzed, that is shown in Table 5. There was statistically significant difference in PCOS women between various genotypes and parameters of hyperandrogenism like FG score and alopecia (p < 0.05).

**Table 5:** Association of *CYP19* gene polymorphism with various hyperandrogenic parameters in PCOS women.

		GG Genotype	AG Genotype	AA Genotype	χ^2^	p value
FG Score	< 8 (n = 72)	18	32	22	6.1129	0.047*
	> 8 (n = 322)	100	165	57		
ACNE	Presence (256)	82	125	49	1.56	0.458
	Absence (138)	36	72	30		
ALOPECIA	Presence (198)	65	103	30	6.191	0.0452*
	Absence (196)	53	94	49		

*Significant p value < 0.05.

## Discussion

Hyperandrogenism or increased androgen production is a considered as key feature in women with PCOS. It has been reported that sisters of PCOS women also have increased levels of androgens, implying that hyperandrogenism may be determined genetically. It is hypothesized that the augmented androgen production in PCOS is a consequence of dysregulation of various genes involved in steroid hormone synthesis^22^. A number of genes have been explored for their association with PCOS. CYP genes code for the essential enzymes in the steroid biosynthesis pathway and are thought to be possible candidate genes in the pathophysiology of this disease and their genetic variants are proposed to be involved in the development of the hyperandrogenism in PCOS.

*CYP19* gene codes for aromatase enzyme which is responsible for converting androgens into estrogens. A number of SNPs of the *CYP19* gene have been studied. Among those, SNP rs2414096 of *CYP19* gene have been widely studied to see any association with PCOS and androgen concentrations, but with variable outcomes. Few studies carried in India also show contradictory results and no study regarding this SNP has been carried in Kashmiri population. This is the first study to examine the possible role of SNP rs.2414096 of *CYP19* gene and its association with PCOS and features of hyperandrogenism in Kashmiri women.

Our results demonstrated a significant difference in genotypic as well as allelic frequencies of *CYP19* gene between PCOS women and non-PCOS group. The frequency of genotype GG, AG and AA in PCOS women was 29.9%, 50% and 20.1% respectively and it was 17%, 53.3% and 29.7% respectively in control group. The frequency of the GG genotype in the PCOS patients was significantly higher than that in the control group while the AA genotype was higher in controls than in PCOS patients, thus indicating that women with GG genotype are at higher risk for developing PCOS as compared to AA genotype groups. Further, when the individual allelic frequency was evaluated between cases and controls, we found that the frequency of “G” allele is 54.9% in cases and 43.6% in controls. The frequency of “A” allele was 45.1% in cases and 56.4% in controls. There was a significant difference in the allele frequencies in cases against controls. Consequently, the presence of “G” allele made them more susceptible to developing PCOS and carriers of allele “A” in PCOS affected individuals was less frequent than the control group, hence suggesting that the presence of “A” allele could be linked with aromatase activity responsible for normal androgen levels which can protect the ovaries from developing PCOS. Our results are in accordance with the case–control study carried out by Jia et al., on 684 individuals consisting of 386 PCOS women and 298 controls who reported increased frequency of the mutant homozygous genotype (AA) in non-PCOS women over the PCOS patients suggesting protective nature in Chinese population^13^. Similar to our results, a study by Hemini et al., and Mehdizadeh et al., also found a significant variation in genotypic and allelic frequencies in SNP rs.2414096 of *CYP19* between the case and control groups and concluded that this SNP is associated with the pathogenesis of PCOS^15, 16^. Similar results were observed by Mutib et al., who reported a positive association of rs2414096 polymorphism of *CYP19* gene with hyperandrogenism and PCOS in Iraqi women^17^. Another study carried on Egypt population demonstrated that genotype AG was significantly high in PCOS Egyptian women compared to controls and found a significant association of SNP with PCOS^14^. Only one study carried in India by Reddy et al., showed association of rs.2414096 polymorphism of *CYP19* with PCOS^21^. However, contrary to our results, a study on Indian population carried out by Ratneev et al., could not find any significant association of *CYP19* rs2414096 polymorphism with PCOS^22^. Table 6 summaries all the previous association studies of rs2414096 polymorphism in *CYP19* gene with PCOS and comparison with the present study.

**Table 6:** Comparison of the present study with previous studies of SNP rs2414096 polymorphism of *CYP19* gene and Polycystic ovary syndrome.

Author	Year	Ethnicity/country	Case/controls	Association with PCOS
Petry^11^	2005	Barcelona/Spain UK	186 PCOS/71 Controls 109 Controls	Yes
Jin Jia^13^	2009	Asian/Han Chinese	386 PCOS/298 Controls	Yes
Hemini^15^	2014	Asian/UAE	62 PCOS/60 Controls	Yes
Reddy^21^	2015	Asian/India	249 PCOS/257 Controls	Yes
Mutib^17^	2015	Asian/Iraq	84 PCOS/65 Controls	Yes
Mostafa^14^	2016	Asian/Egypt	30 PCOS/30 Controls	Yes
Mehdizadeh^16^	2017	Asian/Iran	70 PCOS/70 Controls	Yes
Kaur^22^	2018	Asian/India	250 PCOS/250 Controls	No
Nasira Munawar^18^	2020	Asian/Pakistan	204 PCOS/100 Controls	Yes
Priya^23^	2020	Meta analysis	1414 PCOS/1276 Controls	Yes
Present study	2021	Asian/Kashmiri	394 PCOS/306 Controls	Yes

Further we carried out the correlation analysis of promoter variant of *CYP19* with various parameters of hyperandrogenism. We found a significant correlation between the genotypes and hyperandrogenism among our study population. PCOS women with GG and AG genotype exhibited significantly higher levels of total testosterone and androstenedione as compared to AA genotype and the difference between them was statistically significant. Also, PCOS women with GG genotype had higher mean FG score and hirsutism. These results are consistent with the findings of Sowers and colleagues who studied the relation of rs2414096 SNP of *CYP19* gene with testosterone in PCOS women and found a positive association^12^. A study by Petry et al., in two different populations from Barcelona and Oxford found that variation in aromatase gene is associated with features of hyperandrogenism^11^. Similarly, Mutib et al., reported that PCOS women with GG genotypes had significantly higher mean FG Score and higher level of androgens like testosterone and androstenedione^17^. However, a study by Jia et al., failed to demonstrate any difference in testosterone levels among GG, AG ad AA genotypes of rs2414096 of *CYP19* gene^13^.

## Conclusion

The current study was the first to assess the role of rs 2414096 polymorphism of *CYP19* gene in PCOS women and its association with various manifestations of hyperandrogenism in Kashmiri women. We found that rs 2414096 polymorphism of *CYP19* gene is associated with PCOS as well as with features of hyperandrogenism. The androgen levels, FG Score and alopecia was significantly increased in presence of wild allele than variant allele suggesting that *CYP19* variant alleles imparts a protective role in ovary as well as on symptoms of hyperandrogenism, a feature milieu of PCOS, thus indicating that wild allele may be involved in endocrine abnormalities in Kashmiri women with PCOS.

## Methodology

### Subjects

A total of 700 subjects were recruited for this case–control study which included 394 PCOS and 306 control women. All the participants were age matched, ethnic Kashmiris living in Kashmir province. The approval for the present study was sought from Institutional Ethical Committee (IEC); Sher-e-Kashmir Institute of Medical Sciences (SKIMS), Srinagar, Jammu and Kashmir, India and informed consent was obtained from each study participant.

Women with PCOS were recruited from outpatient clinics of Department of Endocrinology, Sher-e-Kashmir Institute of Medical Sciences (SKIMS), Srinagar, Jammu and Kashmir, India. PCOS was diagnosed according to Rotterdam criteria i.e. having any 2 of the 3 features: (1) Irregularity in menstrual cycles (cycles ≥ 45 days or < 8 cycles per year) (2) Clinical and/or biochemical signs of hyperandrogenism (FG score ≥ 8 or serum total testosterone ≥ 50 ng/dL) and (3) Polycystic ovaries (either ovarian volume of > 10 mL or 12 or more follicles measuring 2–9 mm in diameter)^21^. Women suffering from any endocrinological abnormality like diabetes, Cushing’s syndrome, non classic adrenal hyperplasia (NCAH), hyperprolactinemia, thyroid disorder and androgen-producing tumors were excluded from the study. All the control women were age-matched and were recruited from various Departments of University of Kashmir, Srinagar, Jammu and Kashmir, India. The inclusion criteria for all the controls included females having regular menstrual cycle, no signs of hyperandrogenism and not taking oral contraceptives pills from last six months. All subjects were ethnic Kashmiris and not received hormonal therapy for atleast 3 months before hormonal assay.

### Ethics statement

This study was approved by the Institutional Ethics Committee, Sher-i- Kashmir Institute of Medical Science, Srinagar. Also, all the experiments performed were in agreement with the relevant guidelines and regulations.

All the women fulfilling the diagnostic criteria for PCOS as well as controls were informed about the study. Subjects were recruited after written informed consent was obtained from them.

### Anthropometric and clinical assessment

All the participants underwent a detailed clinical history like age of menarche, presence of acne, alopecia, hirsutism and menstrual history. Hirsutism was graded according to modified Ferriman–Gallwey score that counts nine specified body areas. A score more than 8 was taken as significant. Assessment of anthropometric parameters like height, weight, waist and hip circumference was done to calculate Body mass index (BMI) and Waist-hip-ratio (WHR). BMI was calculated as weight (kg) divided by height squared (m^2^) (BMI = weight (kg)/Height (m^2^). Waist-hip-ratio (WHR) was calculated by dividing the waist circumference (WC) by the hip circumference (HC). Women with PCOS as well as healthy controls were subjected to transabdominal ultrasonography (USG) in order to record typical features of PCO i.e. multiple number of tiny cysts in the periphery of ovary and/or increased ovarian volume.

### Biochemical and hormonal assessment

The peripheral blood sample was collected after 12 h overnight fast on 2nd–3rd day of the spontaneous menstrual cycle or withdrawal bleeding with progesterone in women with amenorrhea. The blood sample required for hormonal and biochemical investigations was collected in clot activated vials whereas for DNA extraction, the blood sample was collected in anti-coagulative Na_2_EDTA vials, which was stored at − 80° for further use. The serum was collected after centrifugation and biochemical and hormonal investigations were done. The hormonal profile included Luteinizing hormone (LH), Follicle stimulating hormone (FSH), Testosterone, 17-hydroxy progesterone (17-OHP)- to rule out non classical congenital adrenal hyperplasia, Thyroid function test (TFT)—to rule out thyroid dysfunctioning, Prolactin (PRL)—to rule out prolactinemia. All the measurements were done by Radioimmunoassay (RIA) on Beckman coulter UniCelDxl 800(Access Immunoassay system) using RIA kits (Immunotech s.r.o, Prague, Czech Republic).

Androstenedione, dehydroepiandrosterone sulfate (DHEAS), Sex hormone-binding globulin (SHBG) and fasting insulin measurements were done by Enzyme-linked immunosorbent assays (ELISA) using Calbiotech, CA USA and DGR Instruments GmbH Marburg ELISA kits on Thermo Scientific Multiskan FC ELISA reader. Insulin resistance was evaluated by three ways—Homeostasis model assessment of insulin resistance (HOMA-IR), quantitative insulin sensitivity check index (QUICKI) and fasting glucose to fasting insulin ratio (FGIR). The formulas used to calculate these risk factors are given as:

The homeostatic model assessment of insulin resistance (HOMA-IR) index was calculated using the formula

$$HOMA = \frac{{Fasting\,Glucose~\left( {{\text{mg}}/{\text{dL}}} \right) \times Fasting~\,Insulin~\left( {\mu {\text{IU}}/{\text{mL}}} \right)}}{{405}}.$$

The quantitative insulin sensitivity check index (QUICKI) was done using the formula

$$QUICKI = \frac{1}{{\log \,Fasting~\,Insulin~\left( {\mu {\text{IU}}/{\text{mL}}} \right) + \log \,Fasting~\,glucose~\left( {{\text{mg}}/{\text{dL}}} \right)}}.$$

The FGIR values were calculated as

$$FGIR = \frac{{Fasting~\,glucose~\left( {{\text{mg}}/{\text{dL}}} \right)}}{{~Fastimg\,~insulin~\left( {\mu {\text{IU}}/{\text{mL}}} \right)~~~~~~}}.$$

The free androgen index (FAI) was derived using the formula:

$$~~~FAI = \frac{{Total\,~Testosterone\left( {{\text{nmol}}/{\text{L}}} \right)}}{{SHBG~\left( {{\text{nmol}}/{\text{L}}} \right)}} \times ~100.$$

The biochemical parameters assessed include oral glucose tolerance test (OGTT), cholesterol, triglyceride (TG), High Density Lipoprotein cholesterol levels (HDL), Low-Density Lipoprotein cholesterol levels (LDL), Urea, Creatinine, ALT, AST. The OGTT was performed by measuring fasting glucose after an overnight fast followed by 2 h post 75 g of oral anhydrous glucose in 300 mL of water. Estimation of all the biochemical parameters was carried out using Erba bioassay diagnostic kits on semi-automatic analyzer (Erba Chemtouch 7, Biochemistry Analyzer, Wiesbaden, Germany).

### Polymorphism genotyping analysis

Genomic DNA was isolated from peripheral blood leukocytes by using QIAamp DNA mini kit (QIAGEN, Hilden, Germany)^24^ and genotyping for polymorphisms of *CYP19* was carried on.

Genotyping of rs2414096 polymorphism of the *CYP19* gene was analyzed by polymerase chain reaction-restriction fragment length polymorphism (PCR–RFLP) method. The PCR product of 189 bp was amplified by using the following primers: forward 5′-TCT GGA AAC TTT TGG TTT GAG TG-3′ and reverse 5′-GAT TTA GCT TAA GAG CCT TTT CTT ACA-3′^13^. The PCR amplification consisted of an initial denaturation at 94 °C for 5 min followed by 35 cycles, each cycle with denaturation at 94 °C for 30 s, annealing was carried out at 48 °C for 30 s and extension was carried out at 72 °C for 40 s, and final extension was carried out at 72 °C for 7 min using thermocycler (Eppendorf, Germany). The PCR products of 189 bp were then subjected to restriction fragment length polymorphism (RFLP) with restriction enzyme CviAII at 37 °C overnight. This digested product of 189 bp was then separated by electrophoresis using 3% agarose gel. The wild type genotype (GG) was not digested yielding a single band of 189 bp; the heterozygote genotype (GA) yielded 3 bands of 189, 161, and 28 bp and homozygous mutant (AA) yielded two fragments of 161 and 28 bp.

### Statistical analysis

Data was managed by Microsoft excel and the baseline quantitative variables were expressed as mean ± SD. All the anthropometric, biochemical and hormonal parameters of PCOS and controls and genotype groups were compared by unpaired student t-test. All the non-parametric variables were calculated by Chi square test. Chi-square (χ^2^) test was used to find out differences in genotype and allele frequencies and test deviations of genotype distribution from Hardy–Weinberg equilibrium between PCOS and controls. Odds ratio and 95% confidence intervals were calculated to test relative risk of dominant, recessive and additive models. One way analysis of variance (ANOVA) was used to compare multiple groups in log additive genotype model followed by Tukey HSD test for intergroup association. The statistical analysis was carried out using statistical computation website vassarstats (http://vassarstats.net/). P-value of less than 0.05 was considered to be statistically significant.

### Ethics approval

This study was approved by the Institutional Ethics Committee, Sher-i-Kashmir Institute of Medical Science, Srinagar under Ethical approval no. IEC-SKIMS Protocol-RP-73/2016.

### Consent of participation

All participants were recruited after written informed consent was obtained from them.

### Consent for publication

All authors have approved the manuscript for submission.

## Data Availability

The data and materials will be provided by authors on reasonable request.

## Abbreviations

17-OHP: 17-Hydroxy progesterone
ALT: Alanine aminotransferase
ANOVA: Analysis of variance
AST: Aspartate aminotransferase
BMI: Body mass index
DHEAS: Dihydroepiandrostenedione sulphate
ELISA: Enzyme-linked immunosorbent assays
FAI: Free androgen index
FG SCORE: Ferriman Gallwey Score
FGIR: Fasting Glucose Insulin Ratio
FSH: Follicle stimulating hormone
HDL-C: High density lipoprotein
HOMA-IR: Homeostasis model assessment-insulin resistance
LDL-C: Low density lipoprotein
LH: Luteinizing hormone
LH:FSH: Luteinizing hormone-follicle stimulating hormone ratio
NADPH: Nicotinamide adenine dinucleotide phosphate
NCAH: Non classic adrenal hyperplasia
PCOS: Polycystic ovary syndrome
PRL: Prolactin
QUICKI: Quantitative Insulin Sensitivity Check Index
RIA: Radioimmunoassay
SHBG: Sex hormone binding globin
SKIMS: Sher-e-Kashmir Institute of Medical Sciences
SNP: Single nucleotide polymorphism
TFT: Thyroid function test
USG: Ultrasonography
WHR: Waist-Hip Ratio
